# How Do PET Myocardial Blood Flow Reserve and FFR Differ?

**DOI:** 10.1007/s11886-020-1274-x

**Published:** 2020-02-12

**Authors:** Nils P. Johnson, K. Lance Gould

**Affiliations:** 1 Weatherhead PET Center, Division of Cardiology, Department of Medicine, McGovern Medical School at UTHealth, 6431 Fannin St., Room MSB 4.256, Houston, TX 77030 USA; 2 Memorial Hermann Hospital, Houston, TX USA

**Keywords:** Positron emission tomography, Myocardial blood flow reserve, Fractional flow reserve

## Abstract

**Purpose of Review:**

This review discusses similarities and differences between cardiac positron emission tomography (PET), absolute myocardial blood flow, and flow reserve with invasive fractional flow reserve (FFR).

**Recent Findings:**

Fundamentally, cardiac PET measures absolute myocardial blood flow whereas FFR provides a relative flow reserve. Cardiac PET offers a non-invasive and therefore lower risk alternative, able to image the entire left ventricle regardless of coronary anatomy. While cardiac PET can provide unique information about the subendocardium, FFR pullbacks offer unparalleled spatial resolution. Both diagnostic tests provide a highly repeatable and technically successful index of coronary hemodynamics that accounts for the amount of distal myocardial mass, albeit only indirectly with FFR. The randomized evidence base for FFR and its associated cost effectiveness remains unsurpassed.

**Summary:**

Cardiac PET and FFR have been intertwined since the very development of FFR over 25 years ago. Recent work has emphasized the ability of both techniques to guide revascularization decisions by high-quality physiology. In the past few years, cardiac PET has expanded its evidence base regarding clinical outcomes, whereas FFR has solidified its position in randomized studies as the invasive reference standard.

## Introduction

During the past decade, coronary revascularization has moved from anatomic-driven decisions to a physiology-based approach. Whereas the COURAGE trial from 2007 [1] selected lesions using a classic, visual 70% diameter stenosis criterion—and failed to provide any benefit over medical therapy—the FAME trial from 2009 [2] treated only lesions with a reduced fractional flow reserve (FFR) and demonstrated superior clinical outcomes. An excess of 2201 citations for the FAME study in 10 years [3] and the elevation of FFR in the guidelines [4] quantify the gradual but relentless transformation. While some countries like the United States have long had a payment criterion that demanded “objective evidence of myocardial ischemia” [5], other countries like Japan introduced specific wording to this effect only in April 2018 [6] in response to the overwhelming accumulation of evidence.

But—often overlooked during the emergence of FFR—cardiac positron emission tomography (PET) with absolute blood flow quantification has shared an intimate connection. Indeed, the second ever publication on FFR [7] validated the metric for the first time in humans via relative flow reserve imaged using cardiac PET. During the 25 subsequent years, several other studies have confirmed that FFR provides an invasive measure of relative stress flow by cardiac PET [8–11]. As such, it might be tempting to assume that cardiac PET and FFR provide completely overlapping physiologic information.

Do FFR and cardiac PET simply offer alternative windows into common physiology? Should they be viewed as interchangeable metrics for informing revascularization decisions? Or do important differences exist between the physiology quantified by these diagnostic techniques? This review addresses these basic yet practical questions in light of emerging research from the past few years coupled with our own clinical experience from using both tools to treat patients in daily practice. In the following sections, we contrast FFR and cardiac PET across a range of categories, highlighting common ground, distinctions, and tradeoffs. Our goal is not to declare a winner except for patients benefiting from thoughtful, comprehensive, and personalized physiologic evaluation to determine the correct diagnosis and rational treatment.

### The Cost of Coverage

#### Invasiveness

Both FFR and cardiac PET expose the patient to a small amount of radiation [12] and the minor risks of vasodilator medications [13–15], making these aspects similar. But FFR requires invasive cardiac catheterization with all its attendant upstream steps including vascular access, anticoagulation, guide catheter placement, iodine contrast injection, and coronary instrumentation. While radial access, smaller sheath and guide sizes, judicious contrast use, and modern 0.014″ pressure wires have reduced the risk, it can never reach the low levels of cardiac PET that only requires insertion of a peripheral intravenous catheter.

Specifically, coronary injury during pressure wire insertion occurs in approximately 0.1% of cases based on a survey of the literature. This rate is somewhat uncertain since many publications do not specifically list or deny adverse, wire-related events during FFR, making ascertainment bias a possibility. A brief survey of the recent literature, reflecting modern catheterization techniques and equipment, identified 8 wire-related injuries among 6181 vessels in physiology protocols [16, 17, 18], including 1 event that eventually proved fatal [18].

Patients presenting for invasive coronary angiography for other reasons—during an acute myocardial infarction or before structural heart interventions, for example—have existing indications to enter the cardiac catheterization laboratory. In these cases, the risk and equipment cost for FFR assessment add trivially to the total procedure. Indeed, skipping immediate FFR assessment for a downstream non-invasive stress test prolonged the hospital stay and increased cost in a randomized trial of patients with unstable angina [19].

#### Chronic Total Occlusions

In the seminal FAME trial, the protocol stated that “because FFR cannot be measured in a totally occluded artery before an intervention is performed, a default FFR value of 0.50 was recorded in the case of totally occluded arteries in the FFR group” [2]. Yet, the physiology is a chronic total occlusion (CTO) and its downstream myocardium varies greatly. As evidence, intracoronary electrographic (ECG) changes during balloon occlusion can be absent down to a collateral pressure index of approximately 0.3 in some patients [20]. This biologic heterogeneity argues against a uniform “FFR = 0.5” default as indicative of the actual intracoronary physiology or necessity of revascularization.

**Fig. 1:**
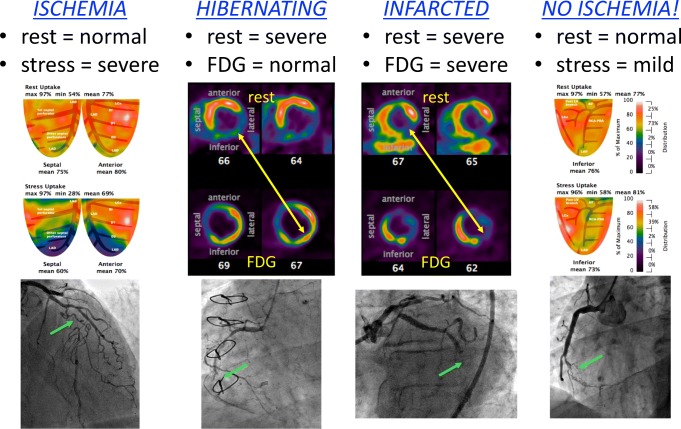
*Chronic total occlusion (CTO) anatomy* versus *physiology*. Because invasive FFR cannot be measured in a CTO before intervention, a default value of 0.50 has been historically assumed. However, cardiac PET distinguishes among four distinct physiologic scenarios, as shown by the examples in which green arrows mark each CTO. Left: the CTO of the mid left anterior descending artery shows normal resting perfusion, indicating viable myocardium, but a large, severe defect during vasodilator stress that would be suitable for revascularization to improve angina. Center left: the CTO of the mid right coronary artery (RCA) has severely reduced resting perfusion but intact glucose metabolism, indicating hibernating myocardium appropriate for revascularization to restore ventricular function. Center right: the CTO of the mid left circumflex artery displays severe and matched reductions in resting perfusion and glucose uptake, indicating a transmural scar without benefit from revascularization. Right: the CTO of the mid RCA has normal resting perfusion, indicating viable myocardium, and only a small, mild defect during vasodilator stress due to robust collaterals from the left coronary artery that prevent ischemia

Furthermore, consider the four cases in Fig. 1, all of which represent CTOs that would have received a default value of 0.50 in FAME. PET imaging identifies that two of them would not benefit from revascularization due to a transmural infarction (matched reduction in perfusion and metabolism tracers) and minimal ischemia (normal perfusion at rest with only mild stress-induced defect). The effort and risk inherent to CTO procedures would only bring advantage for hibernating myocardium of sufficient size (perfusion reduction with intact glucose metabolism) and viable yet ischemic regions (intact resting perfusion with large, severe defect at stress).

Perhaps as a result of these factors of biologic heterogeneity regarding ischemia and myocardial heterogeneity regarding tissue viability, only one of the three multicenter randomized trials of PCI for CTO showed an improvement in symptoms and all of them found no benefit for myocardial infarction or cardiac death [21–23]. Potentially PET-guided selection of CTOs might provide a more appropriate substrate in a future randomized trial, as suggested by PET imaging studies before versus after CTO revascularization [24, 25]. Furthermore, some CTOs are not obvious from the angiogram due to a flush occlusion and minimal collaterals, and therefore, their existence, location, and physiology can only be identified from a PET perfusion image.

#### Extent and Effort

Due to the intrinsic nature of the procedure, PET always images blood flow over the entire left ventricle (LV) whereas FFR studies a single vessel at a time. While FFR can be performed in multiple vessels, in practice, it remains the exception. In a large Japanese real-world registry, multivessel evaluation accounted for only 18% of the cohort [16]. Similarly, a global FFR registry noted multivessel evaluation in just under 27% of patients [26]. While the randomized trial FAME mandated multivessel disease in all subjects [2], more typical physiology trials performed multivessel FFR in only 39% [27] and 41% [28] of subjects.

A rate of 20–40% multivessel FFR evaluation from a wide literature indicates that its routine use most often interrogates just a single vessel. Because studies of mandatory multivessel FFR evaluation demonstrate significant changes in patient management [29, 30], we must assume that occult FFR positive vessels presumed negative, and unexpected FFR negative vessels assumed positive, exist with regularity. Such physiologic surprises in both directions will always be imaged with cardiac PET due to its natural and comprehensive LV coverage.

In addition to the small but non-zero risk of wire-related injury with FFR and the practical drawback to instrumenting multiple vessels, some tortuous vessels produce an “accordion” or “concertina” artifact when straightened by a wire [31]. This folding of the artery produces an iatrogenic pressure gradient from the pseudolesion that precludes valid intracoronary pressure assessment. Cardiac PET, however, images a valid and unperturbed map of myocardial flow over the entire spectrum of epicardial anatomy.

On the other hand, invasive FFR provides millimeter-level resolution regarding the location of pressure changes along the major epicardial vessels when used to create a pullback curve [32]. While a “pullback” (longitudinal gradient) by cardiac PET quantifies and predicts reduced distal invasive FFR [33], PET images have lower spatial resolution than FFR and therefore PET does not distinguish serial stenoses as sharply.

#### Mass and Misses

An important distinction exists between coronary flow (volume per unit time) and myocardial perfusion (flow per mass of tissue). Due to differences in supplied myocardial mass among patients for many reasons (hypertrophy from hypertension, coronary dominance, donor supply to recipient CTO, prior infarction, and male sex [34] probably due to body habitus), comparing flow provides less clarity than comparing perfusion. By nature of its flow model, cardiac PET quantifies myocardial perfusion in cc/min/g, thereby taking the distal mass into account. Similarly, FFR somewhat accounts for the amount of supplied myocardium [35] since pressure gradients depend on volumetric flow that scales with mass [36]. However, FFR does not explicitly quantify downstream mass at risk important for interventions since an FFR ≤ 0.8 in a large artery carries greater clinical weight than the same FFR for a small artery.

A tool for daily practice must perform robustly with rare failures. In the FAME trial, only 27 among 1414 lesions could not be assessed for technical reasons, implying failure in only 1.9% [2]. In two large series, cardiac PET failed in 4 of 208 (1.9%) subjects due to claustrophobia or technical issues [37], similar to 40 failures in 5373 (0.7%) consecutive cardiac PET cases from a routine clinical practice [38••]. Thus, either tool can be successfully applied in 98% or more of cases.

### Peeling the Onion

Coronary stenosis does not take an equal toll on the layers of the myocardium. As well described by perfusion experiments in animals using microspheres [39, 40], the subepicardium experiences a much more mild decrease than the subendocardium in response to epicardial constriction. Indeed, the “decrease in subendocardial and increase in subepicardial flow [are] often associated with normal or even elevated total coronary blood flows so that under the circumstances of these changes, methods that measure only total left ventricular flow … give limited information” [41]. Animal experiments after a transient coronary occlusion demonstrate that subepicardial flow significantly precedes subendocardial reperfusion [42], reflecting the vulnerability of the inner myocardial layer.

Heterogeneity across the myocardium arises from the differential pressures affecting the epicardium (low and steady pericardial pressures largely reflecting intrathoracic pressure) and the endocardium (lower ventricular pressure during diastole but much higher pressure during systole). As a result, subendocardial pressure can equal or even exceed the pressure in the intramural arteries penetrating this layer, producing collapse and cessation or reversal of subendocardial flow [43]. Consequently, subendocardial perfusion becomes limited to diastole, whereas subepicardial flow continues during the entire cardiac cycle. At higher heart rates during exercise, diastole shortens at the same time that myocardial oxygen demand rises, creating the “perfect storm” for subendocardial malperfusion. The observance of angina in patients with aortic stenosis but normal coronary arteries, and its abrupt reversal after aortic valve surgery before regression of LV hypertrophy, reflects this mechanism [44].

#### Imaging Subendocardial Hypoperfusion

The spatial resolution necessary to distinguish subepicardial from subendocardial perfusion varies among imaging techniques. Clearly cardiac magnetic resonance (CMR) imaging offers the highest resolution currently available in humans, with an animal model reproducing the same transmyocardial perfusion gradient by imaging as simultaneously assessed by microspheres [45]. Cardiac PET has a lower resolution than CMR but can still identify transmural gradients. Work in normal subjects using oxygen-15 cardiac PET showed a subendocardial-to-subepicardial perfusion ratio of 1.12 during hyperemia [46]. Under conditions of epicardial stenosis, the same investigators [47] imaged a fall in this ratio to 0.97 when the FFR was intact (> 0.8, indicating minimal to mild epicardial disease) and fell further to 0.88 with reduced FFR (≤ 0.8, indicating moderate to severe epicardial disease).

**Fig. 2:**
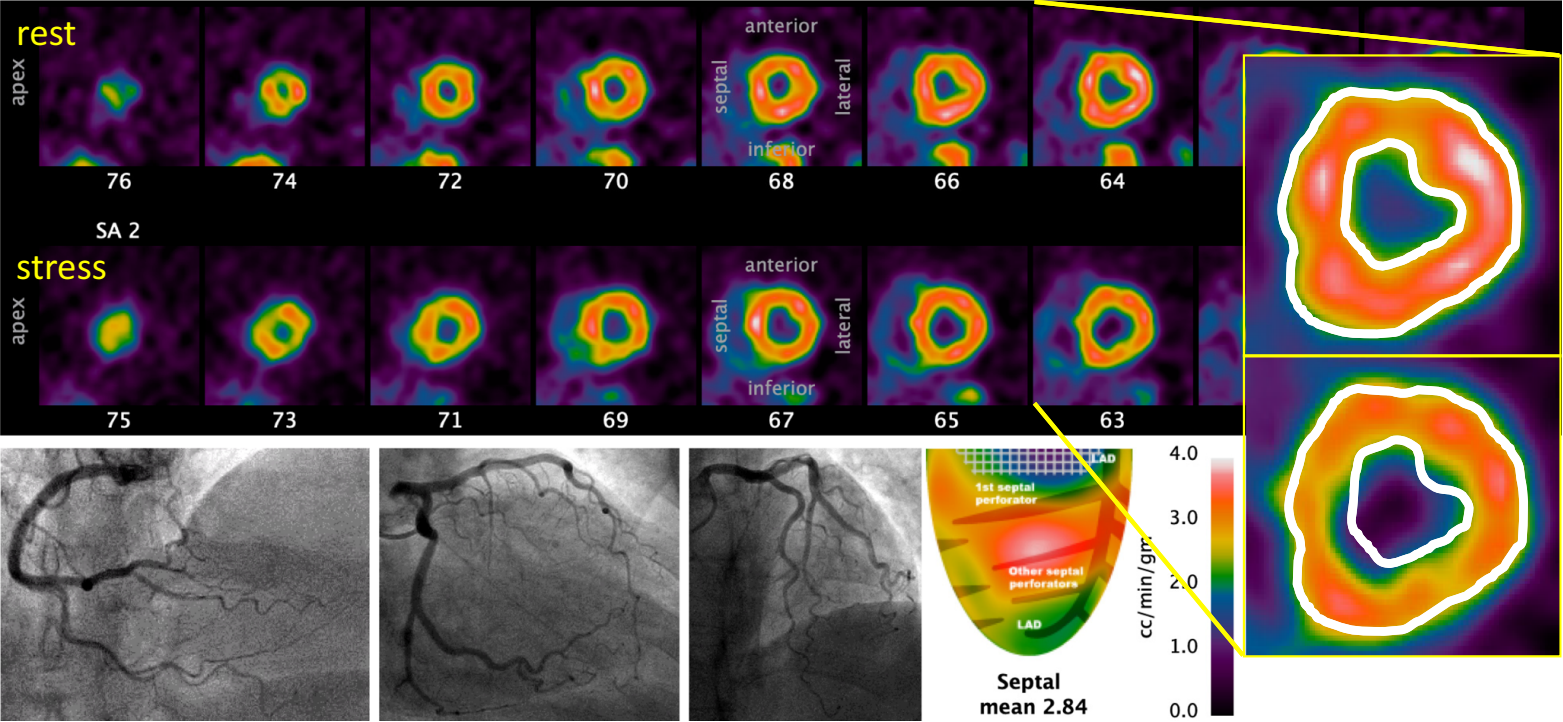
*Subendocardial angina with non-obstructive epicardial arteries*. This 68-year-old woman suffered from exertional angina and dyspnea for several years, prompting an invasive angiogram that demonstrated at most mild atherosclerosis. Echocardiography demonstrated normal left ventricular (LV) function and LV hypertrophy but no significant valvular disease. Due to persistent symptoms, she underwent a cardiac PET scan with dipyridamole stress during which she developed severe angina. An excellent average stress flow of over 2.8 cc/min/g excludes microvascular dysfunction. While no regional defect is present, the inset images copy the resting epicardial and endocardial borders onto the stress tomographic images. A nearly circumferential subendocardial perfusion defect explains her symptoms and would not have been identified by epicardial assessment of FFR, flow reserve, or myocardial resistance

Figure 2 displays a case example from our own clinical experience of a woman with persistent exertional dyspnea and angina but only mild coronary disease at invasive angiography. Dipyridamole hyperemia produced severe angina with 2 mm of ST segment depression without an obvious regional defect on tomographic images. However, inspection of the epicardial and endocardial borders revealed an explanatory, diffuse subendocardial perfusion defect. In her case, LV hypertrophy documented by echocardiography received reasonable flow during vasodilator stress of 2.9 cc/min/g, excluding microvascular disease, but with maldistribution to the subendocardium. Tailored, physiology-guided treatment in her case focused on control of blood pressure for regression of LV mass coupled with agents that slow the heart rate and increase diastolic perfusion time to the subendocardium.

#### FFR in Subendocardial Hypoperfusion

As quantified by two, independent studies using different imaging techniques, FFR has a weak relationship with transmyocardial perfusion gradients [47, 48]. A scatterplot of FFR versus the transmyocardial perfusion gradient measured by CMR showed only a modest correlation coefficient of *r* = 0.63 [48]. Similarly, the scatterplot when assessing transmyocardial perfusion by cardiac PET showed a correlation coefficient of *r* = 0.12 [47], indicating a very weak relationship. Due to this imperfect correlation, cases will often arise with a high FFR yet subendocardial hypoperfusion. These clinical data support the statement from the animal work quoted previously that measurements of total or average transmural LV perfusion (like epicardial FFR) cannot disentangle subepicardial from subendocardial perfusion. For the case in Fig. 2, invasive epicardial evaluation with pressure or flow techniques would not reveal this subendocardial mechanism causing her symptoms. Although not measured explicitly, Fig. 2 would have had a high FFR that would correctly indicate no benefit from revascularization, but miss the mechanism for symptoms by its inability to measure maldistribution across the LV wall.

Along the range of mixed segmental and diffuse CAD from CTO to this example, FFR and PET may be concordant or discordant, both revealing true coronary pressure/flow behavior that should not be viewed as competitive methodology but rather as integrated, individualized, coronary physiology to guide thoughtful, informed management for each patient.

### Flow Versus Pressure

FFR was developed as a relative flow reserve, namely hyperemic flow with stenosis relative to the maximum potential flow in the same artery without stenosis [49]. In the setting of single vessel disease, this relative flow can also be calculated by taking the ratio of hyperemic flow in the stenotic vessel to normal flow in a reference vessel [7]. Indeed, it was by this method that FFR was validated initially by PET in humans [7] after its development in an animal model [49]. Subsequently, a large literature has replicated the linear relationship between relative flow reserve by cardiac PET and invasive FFR [7–11].

Despite this average agreement, close inspection of FFR versus relative flow reserve scatterplots uncovers individual cases of discordance. Namely, some lesions display a large epicardial pressure gradient (and hence a low FFR) but no relative or absolute flow defect. Conversely, other lesions produce minimal pressure gradient (and hence a high FFR); yet, imaging reveals a relative or absolute flow defect.

#### Case Example

**Fig. 3:**
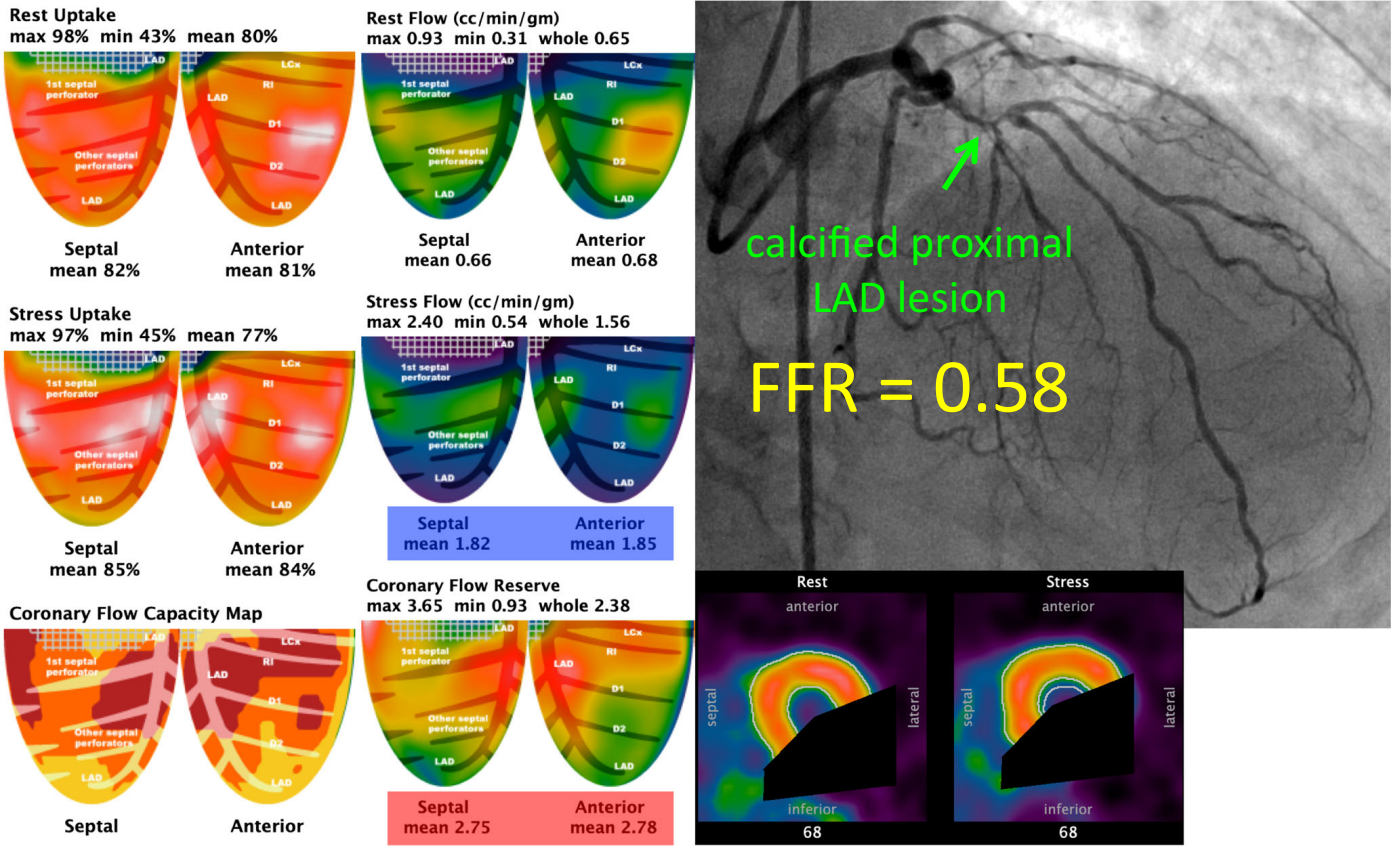
*Absolute* versus *relative flow*. This 78-year-old man had mild angina from a culprit lesion in the left circumflex, not shown since the focus is on the physiology of the left anterior descending (LAD) artery. The anterior and septal quadrants show no relative defect, either for peak uptake or transmural perfusion. Average hyperemic flow exceeded 1.8 cc/min/g, far above the thresholds for myocardial ischemia. Coronary flow reserve was over 2.7, excluding microvascular disease. Wall motion in these quadrants was normal and serum caffeine was negative. Despite intact flow to the LAD distribution, invasive angiography showed a severe, calcified lesion in the proximal vessel with an invasive FFR of 0.58. After revascularization for the angina culprit in another vessel, including a mammary artery bypass of the LAD, repeat cardiac PET showed an increase in flow to 2.8 cc/min/g, indicating a 1.8/2.8 = 0.64 relative reduction in flow similar to the pre-revascularization FFR prediction. In this case, relative flow indeed decreased by approximately 40% due to this lesion, but started from such a high level that its reduction did not produce ischemia, only a pressure gradient

Consider the case in Fig. 3. This patient had a culprit lesion for his mild angina in another vessel, but let us focus on the left anterior descending (LAD) artery exclusively. Cardiac PET measured an average coronary flow reserve (CFR) over 2.7, thereby excluding microvascular disease. Serum caffeine was negative. Mean hyperemic flow exceeded 1.8 cc/min/g, far above the threshold for ischemia [50]. Relative uptake images showed neither transmural nor subendocardial perfusion defect. Regional left ventricular function remained normal. Therefore, by all objective criteria, the LAD had sufficient flow and no ischemia. However, invasive angiography for the culprit in another vessel uncovered an angiographically severe lesion with an FFR of 0.58 in the proximal LAD.

To resolve this apparent discordance, recall that FFR provides an index of the relative—but not absolute—reduction in flow. In this case, after the patient underwent bypass surgery including a mammary graft to the LAD, flow in the LAD by cardiac PET increased from 1.8 to 2.8 cc/min/g, a 1.8/2.8 = 0.64 relative reduction similar to the pressure-based FFR of 0.58. Thus, flow had indeed decreased to approximately 60% of its maximum value, but started from a very high maximum such that the 40% relative reduction failed to reach ischemic thresholds, even in the subendocardium. For most patients, a 25% or greater relative reduction in flow is associated with at least 1 abnormal non-invasive test that returns to normal after revascularization [51]. But Fig. 3 reminds us that this group behavior does not hold for everyone, where the exceptions reveal true pressure/flow physiology essential for optimal, individualized management.

#### Pressure Gradient Versus Perfusion Defect

Further evidence for the lack of universal “ischemia” with a 20–25% relative reduction in flow comes from the CMR literature. Using stress CMR as the reference, only 17% of lesions with a so-called “gray zone” FFR between 0.75 and 0.82 had a major perfusion defect while only another 7% had a minor perfusion defect [52]. That a minority of lesions with FFR values just below the 0.80 threshold commonly used to trigger revascularization produces CMR perfusion defects perhaps explains the results of the recent MR-INFORM randomized trial [53•]. This study found equivalent clinical outcomes and freedom from angina between FFR-guided and CMR-guided revascularization, but with a lower rate of index revascularization when using CMR (35.7% versus 45.0%). Likely, the almost 10% difference in revascularization was driven by lesions with a low FFR but intact CMR perfusion whose revascularization provided no clinical advantage.

### Repeatable Outcomes

#### Test/Retest Repeatability

Every test in clinical medicine carries imprecision due to the test itself as well as temporal, biologic variation. Immediate repeatability on the same day arises mainly from the test mechanics (equipment, assay, sensor), whereas short-term repeatability a few weeks apart also includes biologic variation.

Two large studies using modern pressure wires have shown that invasive FFR has basically no bias (average FFR difference < 0.01) and an imprecision of 0.02 (standard deviation of FFR differences) when measured a few minutes apart [54, 55]. Essentially, all studies of FFR have focused on this immediate retesting, since repeating invasive catheterization over longer periods remains prohibitive in stable patients without intervention. Hence, the short-term test/retest of FFR has not been quantified.

Several investigators have studied both the immediate test/retest of absolute flow by cardiac PET as well its short-term variation within a week or two. The largest study found no bias in paired measurements (all *p* values were not significant) and a same-day variation in absolute stress flow of approximately 10% using rubidium-82 that increased to about 20% variation within roughly 2 weeks [56]. Therefore, 10% variation largely represents the physics of PET imaging and flow quantification, with another 10% biologic variation over the short term.

A metric to compare repeatability among different tests must account for the distinction that absolute flow by PET exists on a ratio scale (0 cc/min/g represents a unique and non-arbitrary state) whereas FFR exists on an interval scale (since an FFR of 0.80 is not “twice as good” as an FFR of 0.40). The coefficient of variation (standard deviation of test/retest repeats divided by their mean value) commonly used to compare analytic test performance requires a ratio scale. Therefore, the ratio scale analog of FFR is the hyperemic pressure gradient (aortic pressure minus distal coronary pressure), equivalent to 1 minus FFR when dividing by the aortic pressure. In this case, a typical FFR value of 0.80 would have a normalized hyperemic pressure gradient of 0.20 so a standard deviation of 0.02 represents 10% for the coefficient of variation. Thus, the immediate test/retest repeatability of both absolute flow by cardiac PET and invasive FFR appears similar.

#### Clinical Outcomes and Cost Effectiveness

A well-established framework exists for evaluating diagnostic tests like cardiac PET and FFR with five criteria: technical accuracy, diagnostic accuracy, clinical pathway, patient outcomes, and cost effectiveness [57]. For the last two criteria, FFR has proven itself in a massive number of randomized controlled trials both against angiography [58, 59] and medical therapy [60•, 61]. These trials have included cost effectiveness analyses, showing it to be cost savings versus angiography [62] and cost effective versus medical therapy [63], although the tradeoff remains sensitive to the actual costs of stent and pressure wire devices that remain dynamic.

On the other hand, cardiac PET perfusion has accumulated a large amount of observational evidence regarding its association with clinical outcomes [38••, 64, 65] but limited randomized comparisons to diagnostic alternatives [66], although it has been used as an imaging endpoint in randomized therapy trials (clinicaltrials.gov NCT00756379) [67, 68]. Economic models, some from clinical trials [69] and others from observational studies [70, 71] or cost analysis [72], have largely suggested cardiac PET perfusion to be cost effective.

While randomized trials of diagnostic testing remain difficult, this challenge applies equally to FFR and cardiac PET. Given the so-called “pyramid of evidence” placing randomized trials above observational data, FFR has appropriately received a stronger level of recommendation versus imaging guidelines that largely do not distinguish among perfusion imaging alternatives [4]. Similarly, cost effectiveness analyses derived from randomized trials carry stronger weight that those derived from observational or simulation sources.

## Conclusions

Health care systems have choices when creating diagnostic pathways for stable coronary artery disease. Currently, many hospitals use clinical symptoms plus traditional testing (treadmill or bicycle exercise electrocardiography, stress echocardiography, stress single-photon emission-computed tomography, or computed tomographic coronary angiography) to select patients for invasive angiography. Three drawbacks to this approach have been clearly identified. First, clinical symptoms associate weakly with the existence or absence of moderate or worse anatomic atherosclerosis, with atypical symptoms actually significantly reducing its prevalence compared to no symptoms [73]; historical pre-test probability prediction scores vastly overestimate current anatomic prevalence [74]. Second, non-invasive testing either is not performed [75], ignored even when moderately or severely abnormal such that only half of such patients are referred to invasive angiography [76], and performs poorly in practice since approximately 40% of patients with angina and a “negative” non-invasive test still have moderate to severe atherosclerosis at angiography [73]. Third, clinical judgment provides only a modest increment in uncovering physiologically obstructive disease from 6% when first ordering a non-invasive test to 27% when proceeding directly to invasive angiography based on physician discretion [77].

Given the weak or modest predictive ability of symptoms and risk scores, non-invasive testing, and clinical judgment, it is not surprising that 39% of stable patients have no or at most mild coronary disease at invasive angiography and another 23% only non-obstructive disease, leaving a minority 38% with obstructive anatomic disease [73]. This low detection rate represents an enormous opportunity to improve patient selection. Two new pathways have been undergoing development.

First, some health care systems have decided to retain their referral practice for invasive assessment but simulate FFR from the angiogram, thereby extending the tool to non-interventional catheterization laboratories plus saving wire costs and avoiding instrumentation risk. A variety of commercial tools has been developed for angiographic-derived FFR with an imprecision of ± 0.07 versus wire-based FFR [78], indicating a marked loss of diagnostic performance for lesions with a true FFR near the 0.75 to 0.80 gray zone [4]. Furthermore, the prevalence of FFR ≤ 0.80 in a pooled analysis of angiographic-simulated FFR in 1842 vessels reached only 34% [78], comparable to FFR ≤ 0.80 prevalence of 34.6% [28] and 36.8% [79] in two recent randomized outcomes trials with a total of 1257 patients. Thus these hospitals must perform approximately three invasive angiograms for one revascularization—a “conversion rate” of only one-third.

Second, other health care systems have invested in upstream “gatekeeper” imaging beyond traditional testing. Already advanced imaging tools like CMR have demonstrated themselves to be equivalent to routine invasive FFR in randomized trials [53•], and simulated FFR from computed tomographic coronary angiography (FFR_CT_) is undertaking an outcomes trial against standard care in the United Kingdom (clinicaltrials.gov NCT03187639). Given fewer restrictions for cardiac PET versus FFR_CT_ and CMR, since it can be performed regardless of renal function, metallic implants, and heart rate and rhythm, more patients can pass through it as gatekeeper and avoid unnecessary invasive angiography, thereby reducing cost and increasing efficiency of the catheterization laboratory. In our own clinical experience, patients we recommend for invasive procedures based on cardiac PET have a “conversion rate” near 80%, with a minority having severe disease without a revascularization target or a CTO whose treatment remains controversial as detailed earlier in this review.

In conclusion, FFR has produced a sea-change in our approach to revascularization, moving the needle from anatomy to physiology. Cardiac PET and FFR provide fundamental tools for modern revascularization of stable coronary lesions, with similarities and differences summarized in Table 1, all reflecting true pressure/flow physiologic variability among individuals needing thoughtful, informed management specific for each patient. In our opinion, patients, physicians, hospitals, and health care systems cannot thrive without either tool.

**Table 1:** Comparison of cardiac PET and FFR among various criteria

	PET	FFR	Advantage
Invasive	No	Yes	PET
LV coverage	Whole	Partial	PET
CTO/tortuous	Yes	No	PET
Flow	Absolute	Relative	pet^a^
Subendocardium	Partial	No	pet
LV mass	Yes	Indirect	pet
Failure rate	< 2%	< 2%	tie
Test/retest (min)	± 10%	± 10%	tie
Test/retest (weeks)	± 20%	Unknown	N/A
Spatial resolution	Good	Excellent	ffr^a^
RCT	Limited	Numerous	FFR
Cost effective	Probably	Yes	FFR

^a^Lowercase “pet” and “ffr” denote a modest advantage whereas upper-case “PET” and “FFR” denote a clear advantage

*CTO* chronic total occlusion, *FFR* fractional flow reserve, *LV* left ventricular, *N/A* not available, *PET* positron emission tomography, *RCT* randomized controlled trial
